# Leveraging FDA Labeling Documents and Large Language Model to Enhance Annotation, Profiling, and Classification of Drug Adverse Events with AskFDALabel

**DOI:** 10.1007/s40264-025-01520-1

**Published:** 2025-02-20

**Authors:** Leihong Wu, Hong Fang, Yanyan Qu, Joshua Xu, Weida Tong

**Affiliations:** 1 Division of Bioinformatics and Biostatistics, National Center for Toxicological Research, U.S. FDA, 3900 NCTR Rd, Jefferson, AR 72079 USA; 2 Office of Scientific Coordination, National Center for Toxicological Research, U.S. FDA, 3900 NCTR Rd, Jefferson, AR 72079 USA

## Abstract

**Background:**

Drug adverse events (AEs) represent a significant public health concern. US Food and Drug Administration (FDA) drug labeling documents are an essential resource for studying drug safety such as assessing a drug’s likelihood to cause certain organ toxicities; however, the manual extraction of AEs is labor-intensive, requires specialized expertise, and is challenging to maintain, due to frequent updates of the labeling documents.

**Objective:**

To automate the extraction of AE data from FDA drug labeling documents, we developed a workflow based on AskFDALabel, a large language model (LLM)-powered framework, and its demonstration in drug safety studies.

**Methods:**

This framework incorporates a retrieval-augmented generation (RAG) component based on FDALabel to enhance standard LLM inference. Key steps include (1) selection of a task-specific template, (2) FDALabel database querying, and (3) content preparation for LLM processing. We evaluated the performance of the framework in three benchmark experiments, including drug-induced liver injury (DILI) classification, drug-induced cardiotoxicity (DICT) classification, and AE term recognition.

**Results:**

AskFDALabel achieved F1-scores of 0.978 for DILI, 0.931 for DICT, and 0.911 for AE annotation, outperforming other traditional methods. It also provided cited labeling content and detailed explanations, facilitating manual verification.

**Conclusion:**

AskFDALabel exhibited high consistency with human AE annotation, particularly in classifying and profiling DILI and DICT. Thus, it can significantly enhance the efficiency and accuracy of AE annotation, with promising potential for advanced AE surveillance and drug safety research.

**Supplementary Information:**

The online version contains supplementary material available at 10.1007/s40264-025-01520-1.

## Key Points

**Table:** 

AskFDALabel incorporates term-of-interest (TOI) recognition and retrieval-augmented generation (RAG) for an enhanced drug adverse event (AE) annotation.
AE extraction through automation with AskFDALabel is highly consistent with the results from the manual curation by experts.
Both drug-induced liver injury and drug-induced cardiotoxicity classification with AskFDALabel achieved a high F1-score above 90%.

## Introduction

Drug adverse events (AEs) are a major cause of death in the United States (US) [1]. Data from the US Food and Drug Administration (FDA) Adverse Events Reporting System (FAERS) reveal that the number of annual AE reported cases associated with “death” has surpassed 70,000 since 2020 [2]. Identifying, profiling, classifying, and monitoring AEs from various drug safety documents, such as drug labeling documents, individual case safety reports (ICSRs), literature, and other data sources, are at the center of current AE research, and are also critical for drug safety review in many regulatory agencies, including the FDA.

FDA drug labeling documents serve as authoritative resources for AE research. These documents, submitted by manufacturer and then approved by the FDA, contain important drug safety information in sections such as “Boxed Warnings,” “Warnings and Precautions,” and “Adverse Reactions.” Due to their comprehensiveness and reliability, these documents are the primary source for researchers and healthcare professionals as a key reference for understanding drugs’ safety profiles [3]. Recently, the FDA National Center for Toxicological Research (NCTR) developed FDALabel [4], a tool widely recognized within and outside the FDA, enabling users to efficiently access and query over 150,000 drug labeling documents.

Over the past decades, FDA drug labeling documents have been used extensively in AE studies. For example, the Side Effect Resource (SIDER) has curated AEs for 1430 marketed medicines, with most sourced from FDA drug labeling documents [5]. Wu et al. systematically analyzed and compared the AE profiles of 1164 drugs by mapping their labeling documents to Medical Dictionary for Regulatory Activities (MedDRA) [6]. Roberts et al. developed a benchmark for AE detection by manually annotating 200 drug labeling documents, which was used for the Adverse Reaction Extraction Challenge at the 2017 Text Analysis Conference (TAC) [7]. Similarly, Bayer et al. manually annotated another 200 drug labeling documents, by focusing on detecting AE terms particularly relevant to FDA reviewers [8].

Additionally, FDA drug labeling documents have been widely used in drug toxicity classification and annotation. For example, during the past two decades, the research team at the NCTR has produced several drug-induced toxicity benchmark datasets by manually reading and analyzing drug labeling documents, such as those for drug-induced liver injury (DILI) [9–11], drug-induced cardiotoxicity (DICT) [12], drug-induced renal injury list (DIRIL) [13], and others, which offer large collections of manually curated drug toxicity annotations. These datasets have spurred numerous artificial intelligence (AI)/machine learning initiatives on drug toxicity prediction [14–18].

Labeling-based AE annotation and profiling by human experts poses three main challenges. First, it typically requires a labor-intensive, time-consuming manual process of extracting and annotating AE data. Frequent updates to labeling documents necessitate regular repetition of this process, making the maintenance of up-to-date AE monitoring an expensive, if not impossible, process. Second, to reduce the cost of this process, some studies applied standardized terminologies like MedDRA for AE extraction, but this often led to missing some critical AEs, as the FDA does not mandate the use of MedDRA terminology in labeling [6, 19]. Last, the manual AE extraction from the labeling documents is expertise-dependent, which could lead to varying results among experts.

Recent advancements in large language models (LLMs) have offered a transformative opportunity to modernize labeling-based AE research. LLMs have the potential to automate and streamline AE term extraction, profiling, and classification. These models can now analyze vast amounts of textual data with high consistency and accuracy, reducing the burden of manual curation and minimizing variation among human experts.

With concerns for security, regulatory agencies such as the FDA have primarily been employing LLMs locally to analyze data. We developed AskFDALabel (version 1) [20], a framework to employ open-source LLMs in a secure environment with a retrieval-augmented generation (RAG) mechanism to enhance the efficiency and effectiveness of drug labeling document analysis for reviewers and research scientists. In the first version, we explored the potential to use locally hosted models (e.g., Llama [21] and Falcon [22]) with RAG to provide more relevant and accurate responses from FDA labeling documents [20]. Additionally, we fine-tuned the employed LLMs using low-rank adaptation (LoRA) and Alpaca strategy [23], which involved automatically generating training content through a larger LLM, such as GPT-3.5.

In this paper, we further improved AskFDALabel as a tool to interface with FDA labeling documents in a study of drug safety. Particularly, we introduced new features in the current AskFDALabel framework (version 2), such as the term-of-interest (TOI) recognition, a database-enhanced RAG process, and special templates. We then outlined the results obtained from three experiments: (1) DILI classification, (2) DICT classification, and (3) drug AE profiling, and evaluate their performance by comparing existing methods, including manual review.

## Material and Method

### Datasets

#### Drug-Induced Liver Injury (DILI) Dataset

We used 287 DILI annotated drugs, retrieved from the original DILI class publication [9]. Among them, 76 drugs were currently labeled as “withdraw” or “discontinued.” Since their drug labeling documents were not searchable in the FDALabel database, they were excluded for this analysis. For the remaining 211 available drugs, we further combined the classifications “most” and “less” DILI concern as DILI positive, where “no” DILI concern is considered as DILI negative. This resulted in 154 DILI-positive and 57 DILI-negative drugs.

#### Drug-Induced Cardiotoxicity (DICT) Dataset

For DICT analysis, we processed 1184 drugs labeled with DICT concern levels—i.e., most, less, or no DICT concern—from a previous publication [12]. We successfully found and processed the labeling for 1167 of these drugs. As with the DILI classification analysis, we combined the most and less DICT concern groups into a DICT-positive category. This resulted in 829 DICT-positive and 338 DICT-negative drugs.

#### Drug Adverse Event (AE) Profiling

To evaluate the performance of the generated AE profile from labeling documents, we processed 200 drugs with AE profiles that had been manually annotated by human in the TAC 2017 challenge [24]. In detail, over 26,000 adverse reaction terms (~13,000 unique terms) were manually annotated by human reviewers in TAC, which is around 70 AEs per labeling document. The whole effort took years to complete, with a group of reviewers from the FDA and National Institutes of Health (NIH) [7]. We conducted the analyses based on both section level (which included 476 labeling sections in total) and the whole drug document level (200 drug labels). For the section level analysis, we used the original labeling content provided by the dataset; for the whole drug document level analysis, we only used the drug name, and the most recent labeling document was automatically retrieved by AskFDALabel during the study.

### Term-of-Interest (TOI) Recognition

We implemented a new TOI recognition feature in the AskFDALabel framework by utilizing an LLM. In a previous study, we designed a simple hard-coded approach to let the user manually annotate the key attribute terms inside the query. For example, instead of the query “What are the adverse events reported for abacavir?”, the user can substitute “What are the adverse events reported for abacavir?” Although such an approach will increase users’ preparation time to input their queries, it can largely improve the accuracy and relevance of the RAG process.

In addition to the hard-coded approach, we also used an LLM-based TOI recognition approach to identify and extract key attribute terms. In contrast to regular named entity recognition (NER) approaches to train a model based on a tagging annotator, the LLM-based approach tried to directly recognize TOIs in a generative style. For example, using the input query “What are the adverse events reported for abacavir?”, the LLM is expected to respond “[Drug-Name] Abacavir” with appropriate prompts. The LLM-based NER prompt used in this step is provided in the electronic supplementary material (Online resource 1).

### Retrieval Augmented Generation (RAG) Process

Most, if not all, RAG technologies are based on the idea of semantic similarity or relevance to the input query to fetch relevant information from a large corpus of text. In general, the reference texts will be segmented into smaller text chunks, where vectorization will be performed to digitalize the text into a one-dimensional, numerical vector. Word embedding approaches such as Global Vectors for Word Representation (GloVe), Sentence–Bidirectional Encoder Representations from Transformers (SBERT), and Doc2Vec are applied in this transformation process [25–27].

In this study, we developed a hybrid information retrieval process, named FDALabel-based RAG, by combining an LLM with a regular database search in the RAG process. This approach has the advantages of regular database searching; by using an appropriate database query, the user will not risk a hallucination in the results. In contrast, an LLM model auto-detects keywords mentioned in the user input and then converts it into the database-search query.

For example, in DILI classification, after the user query identifies the drug of interest, a database query will be generated to retrieve labeling documents that meet certain criteria consistent with the guidance during manual reading. Particularly, the drug must be a human prescription drug (preferred) or over-the-counter (OTC) drug. Single-ingredient drugs are preferred over combined drugs in the query. In addition, we used the most recently revised labeling data. For DILI classification, three labeling sections were considered in the RAG process: “Boxed Warnings,” “Warnings and Precautions,” and “Adverse Reactions.”

After the labeling content was retrieved, the next step was to use the LLM to find DILI keywords from the selected labeling sections. The prompt used in this step is provided in Online resource 1 (see the electronic supplementary material). As a result, the model would return any DILI keywords found in these labeling sections.

Finally, we used the exact criteria for DILI classification mentioned in a previous publication [9] to judge the DILI based on the LLM output.

### Special Templates

Special templates are a novel design in the AskFDALabel framework to enable better handling of distinct user requests in one platform. For example, a query such as “What are the adverse events of abacavir?” relies on a single labeling document; whereas another such as “What are the newly added/reported adverse events of tamoxifen in the past five years?” will require information from multiple labeling documents. To cope with common user requests, we implemented several task-specific templates and designed an LLM-enabled module to automatically determine which special template is the best fit for a given query.

In this study, we used the locally hosted model Llama 3.1-70B to determine whether special templates would be used for the given query, and which one(s). We designed a special template for each experiment: (1) for DILI classification, the template was designed to automate the classification scheme to assess DILI [9] based on the labeling description; (2) for DICT classification, the template was designed to classify DICT [12] based on the labeling description; and (3) for drug AE profiling, the template was designed to generate an AE profile of a given drug summarizing all AEs mentioned in its most recent labeling documents. A typical question that fits into the first template is “What are the adverse events reported for abacavir?” The detailed prompts used to determine the appropriate template to use and prompts for these three templates are provided in Online resource 1 (see the electronic supplementary material).

When a special template was selected, the RAG process used different criteria to get the labeling document and return the information required by each template. In DILI classification, it returned “Boxed Warnings,” “Warnings and Precautions,” and “Adverse Reactions” sections since only these three sections are used in DILI classification. For DICT classification, the above three sections plus the “Overdosage” section were considered, due to the different guidance provided by human scientists in previous publications [12]. For AE analysis, the entire labeling document was used in the RAG process.

### The LLM Core of AskFDALabel

In the most recent AskFDALabel framework, we used Llama 3.1-70B [28], the latest released Llama model at the time, as the LLM core for AskFDALabel. It had two major advantages: (1) The Llama3.1 series supported up to 128,000 input tokens, which enabled us to fit the entire labeling document into the input prompt. The previous Llama model used in AskFDALabel supports only 4000 tokens, which forced the segmentation of the labeling document into multiple text chunks. (2) The 70B model proved to be a significant improvement overall in comparison to the previously tested 8B and 13B models.

All the LLM experiments ran on a graphics processing unit (GPU) server with eight H100 80 Gb GPUs. Four of the eight GPUs were utilized for this study to handle the Llama 3.1-70B model. The model was loaded in fp16 format using vLLM [29].

### Exact and Semantic-Based Matching to Evaluate AE Profiling

**Fig. 1:**
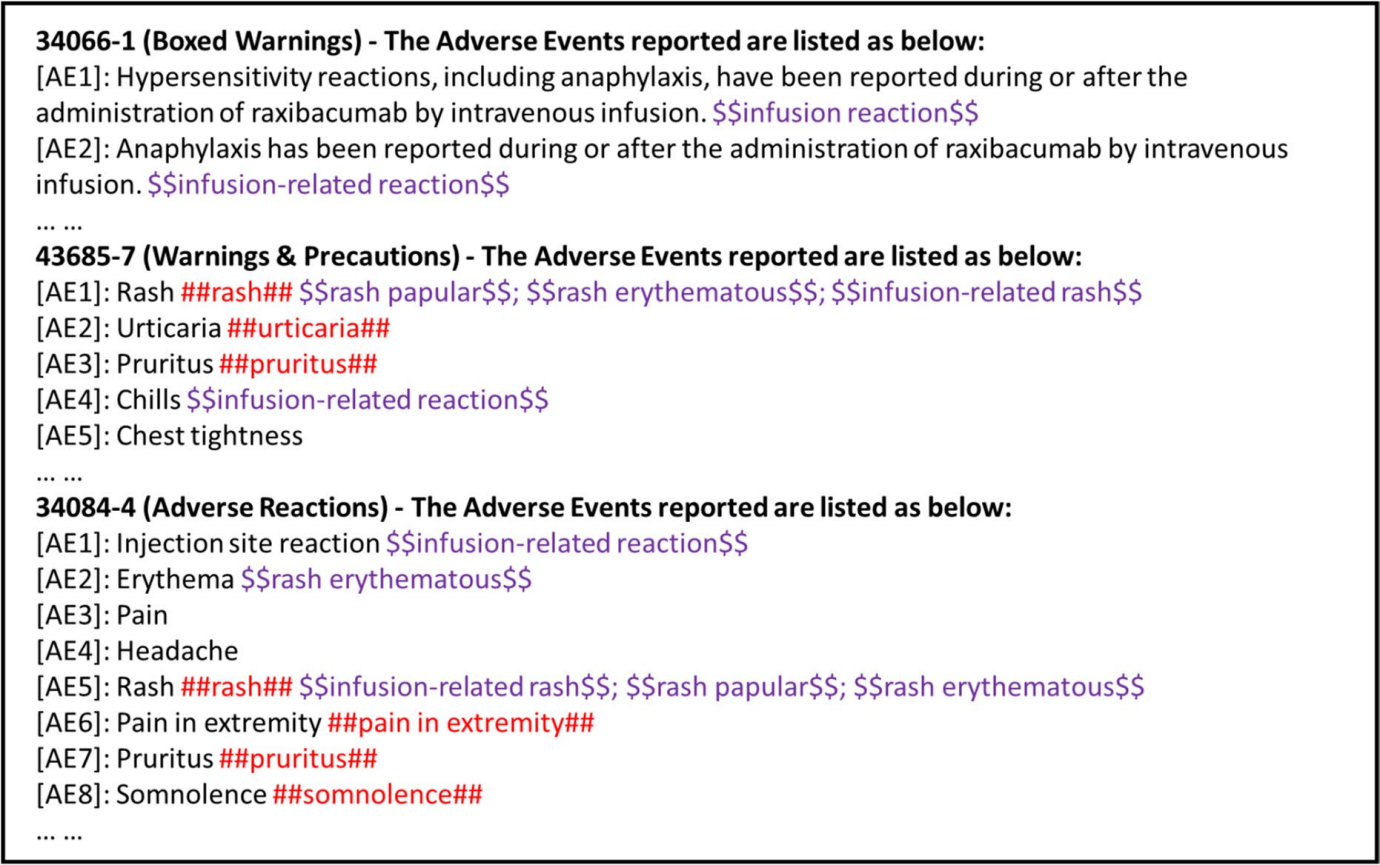
An example of drug AE profiling contents for raxibacumab generated by AskFDALabel. We matched the generated AE profile with human annotation (ground truth) in two ways: (1) hash tags (##) indicated human-annotated AEs were matched exactly in the AE profiling; (2) dollar tags ($$) indicated human-annotated AEs were matched semantically based on LLM determination. *AE* adverse event, *FDA* Food and Drug Administration, *LLM* large language model

To better evaluate the performance of drug AE profiling, we introduced two matching algorithms—exact and semantic-based matching. The exact match only counts the ground truth term that appears exactly in the AskFDALabel response. On the other hand, semantic-based matching will also consider terms that have semantically similar terms in the AskFDALabel response. To implement semantic-based matching, an LLM-based approach was applied to define semantically similar terms between the LLM result and the ground truth (i.e., human reviewer annotations). For an example, see Fig. 1, which illustrates the drug AE profiling contents for raxibacumab generated by AskFDALabel. The hash tags (##) indicate exactly matched terms, and the dollar tags ($$) indicate semantically matched terms. For instance, the exact term “infusion-related reaction” is not found in the generated drug AE profile; however, the profile contains “injection site reaction,” a semantically similar term. Therefore, “infusion-related reaction” will be considered as a semantically matched term, but not as an exactly matched term. The detailed prompts used for semantic matching are provided in Online resource 1 (see the electronic supplementary material).

## Result

### AskFDALabel Framework

**Fig. 2:**
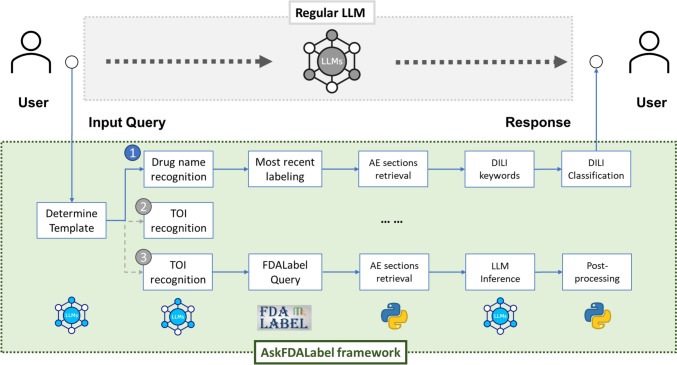
The AskFDALabel framework for drug AE analysis. This framework introduces a structured, end-to-end pipeline that processes user queries through a series of specialized steps, rather than directly using an LLM. Upon receiving a user query, the framework determines the appropriate template to apply based on the query type. For example, in a DILI classification task (*first template in figure*), the framework identifies and extracts the drug name, retrieves the most recent labeling document from the FDALabel database, and extracts AE sections, such as “Boxed Warnings,” “Warnings and Precautions,” and “Adverse Reactions.” The LLM then identifies DILI-related keywords from these sections. A post-processing step evaluates the extracted keywords to assess whether the drug poses a DILI risk. The pipeline’s output is then provided to the user. *AE* adverse event, *DILI* drug-induced liver injury, *FDA* Food and Drug Administration, *LLM* large language model, *TOI* term-of-interest

The overall framework of AskFDALabel is shown in Fig. 2. This framework integrated a RAG component based on FDALabel, enhancing the capabilities of a regular LLM inference pipeline. Key steps in this RAG-based enhancement included (1) template selection, (2) FDALabel database query, and (3) content preparation for LLM inference.

We began the process by identifying the nature of the user query through template selection, which involved determining the focus of the inquiry, (e.g., drug name, AEs, or other entities). Next, we employed a specialized LLM-based inference to select the appropriate processing template. Based on the chosen template, we executed a query on the FDALabel database to retrieve relevant information. Depending on the template requirements, this involved retrieving sections, such as AEs, from a single labeling document, or gathering data from multiple documents. Retrieved information was then organized into a prompt format that the LLM could interpret. If multiple references were gathered, the initial user query (e.g., “What are the adverse events reported for abacavir?”) could be split into sub-queries (e.g., “What adverse events are reported in the Boxed Warnings section for abacavir?”). These sub-queries were processed by the LLM individually, and a final response was compiled from all sub-query outputs.

This integrated framework leveraged FDALabel data to improve the relevance and specificity of responses to user queries, enhancing the standard LLM inference pipeline for applications in AE research and classification tasks such as DILI classification.

### Large Language Model (LLM)-Based TOI Recognition

LLM-based TOI recognition was implemented in the updated version of FDALabel. To evaluate its performance to identify TOI such as drug names, we evaluated its performance in recognizing a drug name from 2141 unique drug trade names from FDALabel that satisfied the following criteria: (1) human prescription, (2) New Drug Application (NDA), (3) remove Repacker and Relabeler, and (4) Reference Listed Drug (RLD). Additionally, we manually validated the result to make sure the synonyms were also considered as correct. For example, drugs Atelvia and Actonel were identified as “risedronate sodium,” the generic name of these drugs. As a result, 2037 drugs (95.14%) were successfully identified by the LLM search, of which 1848 drug names were exactly used in the database query, and the remaining 189 drugs were identified using a semantically similar name, such as using “risedronate sodium” instead of “Atelvia.” With that said, LLM-based TOI recognition can accurately identify and recognize drug names from the free-text style of user inputs. Therefore, we decided to use the LLM-based TOI recognition in the AskFDALabel framework.

### DILI Classification

AskFDALabel was applied to 211 DILI annotated drugs. As the results show, 153 of 154 DILI-positive drugs (99.4%) and 44 of 57 DILI-negative drugs (77.2%) were labeled consistently between the manual curation and our AskFDALabel output. This result outperformed previous Bidirectional Encoder Representations from Transformers (BERT)- and keyword-based DILI classification results, even without the needs of training a predictive model [19, 30].

Moreover, we investigated the 14 mismatched (i.e., 13 false-positive and 1 false-negative) cases between AskFDALabel and manual curation (Online resource 2; see the electronic supplementary material). These 14 cases were manually verified with the following criteria: (1) the drug labeling document used for the AskFDALabel process is valid, as the name on the labeling document matches the given drug name; (2) DILI-related keywords found by AskFDALabel could be manually found in the given reference; and (3) a review of the labeling document could determine whether the discrepancy between the human and AskFDALabel result is due to a labeling document update since the manual reading.

We found that AskFDALabel responses to seven of 13 false-positive cases could be verified, as their references contained DILI-related terms. Among the seven verified cases, four were due to labeling updates. For example, the labeling document for Desfaral (deferoxamine) did not have any hepatic information in 2008 according to the labeling archive from DailyMed, but its labeling was updated in 2010 to include such information. Similarly, the labeling document for ketamine did not mention DILI-related terms before 2020, but a new subsection titled “Drug-Induced Liver Injury” was added in 2021. Metronidazole’s labeling was updated in 2024 to include hepatic information in the “Adverse Reactions” section, which was not present in its previous labeling. For the other three cases, we found keywords such as “alkaline phosphatase increased” or “elevation in liver function tests” to confirm they could be DILI-positive drugs. Validation notes are provided in Online resource 2.

On the other side, clotrimazole, which was determined to have “no” DILI concern by AskFDALabel but “less” DILI by a human scientist, is the only false-negative case reported in this study. A potential reason for this is the latest labeling documents of clotrimazole used by the AskFDALabel framework are for the topically administered product. After consulting with our DILI experts, they agree that it is better to primarily focus on the product that was taken orally or parenterally. Further investigation is needed to see whether a revision of the special template focusing on oral drugs could further improve performance.

By reconsidering these verified mismatched results as correct, only one false-negative drug and six false-positive drugs remained. The updated F1-score was 0.978 (Table 1). That said, we demonstrated that using AskFDALabel can retrieve results that are accurate and highly consistent with those processed by human reviewers. Also, using the automated process, the current framework is capable of capturing the impact on DILI classification resulting from recent changes in the labeling document. The detailed DILI classification results for all assessed drugs are provided in Online resource 3.

**Table 1:** Statistical summary of DILI and DICT classification result

	Approach	Accuracy	Recall	Precision	Specificity	F1-score
DILI classification	AskFDALabel	**0.967**	0.994	**0.962**	**0.895**	**0.978**
	BERT model [19]	0.927	**1.000**	0.784	0.900	0.879
	Keywords [19]	0.822	**1.000**	0.581	0.764	0.735
	XGBoost [30]	0.839	0.651	0.856	0.941	0.740
DICT classification	AskFDALabel	**0.901**	**0.941**	**0.921**	**0.802**	**0.931**
	Prev. LLM [20]	0.776	0.608	0.907	0.940	0.728
	ChatGPT (3.5) [20]	0.715	0.908	0.647	0.531	0.756

Bold value in each column represents the best performance across all approaches for DILI and DICT classification

*BERT* Bidirectional Encoder Representations from Transformers, *DICT* drug-induced cardiotoxicity, *DILI* drug-induced liver injury, *FDA* Food and Drug Administration, *LLM* large language model

### DICT Classification

Similarly, we processed the 1167 DICT drugs, where 780 of 829 DICT-positive drugs (94.1%) and 271 of 338 DICT-negative drugs (80.2%) were correctly classified by AskFDALabel. The statistical measurement is summarized in Table 1. The overall F1-score for DICT classification was 0.931, significantly improving upon results from a previous LLM (Llama 13B) used in AskFDALabel (F1-score = 0.728) and GPT-3.5 (F1-score = 0.756) [20]. This superior performance may be attributed to the use of the newer LLM (Llama 3.1-70B) and the hybrid information retrieval strategy. The complete DICT classification results for all assessed drugs are provided in Online resource 4 (see the electronic supplementary material).

### Drug AE Profiling

In this section, we performed two sub-experiments on the drug AE profiling: (1) We used the original labeling contents provided by the TAC dataset. This enabled us to develop a comparative study for AskFDALabel to compare currently existing approaches within the same dataset. (2) We followed the AskFDALabel framework, using the drug name to retrieve the most recent labeling documents for the AE profiling process. The content of the labeling documents could be updated during the past years and, therefore, may identify newly reported AEs.

Sub-experiment 1 contained 476 data samples, where each sample represented content from one section of the labeling document. To determine the true positives, which were the profiles annotated by both human and LLM, we mapped each line of the AE profiling to human-annotated AEs, and added tags if an AE was matched. Based on the exact match, the recall and F1-score on the section level were 0.825 and 0.827, respectively.

Additionally, we considered semantically similar terms between the AskFDALabel result and the human annotations. As a result, the average semantic-based recall and F1-score for all 476 samples were 0.939 and 0.911, respectively, which were higher than previously reported approaches in the TAC 2017 challenge [7] as well as when using the NER predictive model based on RxBERT [31]. Moreover, our LLM-based approach had another significant advantage in that it did not need to train the NER model with an already annotated dataset.

Next, sub-experiment 2 retrieved the most up-to-date labeling documents based on the drug name. The generated AE profile for each drug was then compared to the true AE profile annotated by reviewers (which was still based on the older version) on the whole labeling level. As a result, 188 drug names were successfully processed and their AE profiles were generated by AskFDALabel. Twelve drugs could not be processed. Some of the drugs, such as “Arcapta” and “Belviq,” had been discontinued since the TAC annotation. Therefore, their labeling information was no longer available in the current FDALabel database.

As summarized in Table 2, the average semantic-based recall for 188 drugs on the whole labeling level was 0.895, and the overall F1-score was 0.859. The decreased performance compared to directly using TAC contents may have been due to the inaccuracy in labeling retrieval. For example, a drug named KIT was incorrectly processed due to its name after examination, but another major reason could be the result of labeling updates during the past decade since the human annotation. In other words, the different AE terms found by AskFDALabel could still be true AEs. For example, “anaphylaxis” was found in the raxibacumab AE profiling by AskFDALabel but not in the TAC 2017 human annotation, due to the label update of raxibacumab on 06/2021; therefore, the annotations by both humans and AskFDALabel were accurate because of the different accessed time of the labeling data. The AE profiling generated by AskFDALabel made it much easier to catch these recent labeling updates related to AE changes.

**Table 2:** Performance on TAC 2017 dataset and drug AE profiling

	Approaches	Precision	Recall	F1-score
Sub-experiment 1: TAC original labeling	AskFDALabel—exact	0.858	0.825	0.827
	AskFDALabel—semantic	**0.906**	**0.939**	**0.911**
	TAC 2017—SOTA [7]	0.851	0.853	0.852
	RxBERT [31]	0.893	0.885	0.889
Sub-experiment 2: most up-to-date labeling	Exact—up to 1024 tokens output	0.758	0.769	0.753
	Semantic—up to 1024 tokens output	0.840	0.895	0.859
	Semantic—up to 4096 tokens output	**0.848**	**0.904**	**0.870**

*AE* adverse event, *FDA* Food and Drug Administration, *TAC* Text Analysis Conference, *SOTA* State-Of-The-Art

Moreover, we investigated the impact of output length on the final performance for drug name only (Table 2). In this study, the output length was set to 1024 tokens per section by default. Although this output length met most of the requirements during our experiment, it was not enough for a few labeling documents that could contain hundreds of AE terms in one section. Therefore, we further increased the output length to 4096, with everything else unchanged. As a result, performance increased slightly compared to that of the 1024 output length, although longer output length also increase the processing time from 160 min to 180 min, for processing 200 drugs. Note that the increase of maximum output would not affect most of the queries since their outputs did not reach the token limits.

In all, we demonstrated that AskFDALabel also generated high-quality AE profiling for a given drug. The detailed AE profiling report of the 188 drugs supporting the above analysis is provided in Online resource 5 (see the electronic supplementary material).

## Discussion

### Improved RAG with Hybrid Information Retrieval

One important feature of AskFDALabel is the utilization of hybrid information retrieval, which combined database query with LLM inference to find the most relevant references for the user query.

Database query has been widely used in past decades and is still the main approach for most information retrieval tasks. While database query has several advantages, the most important, if not the only one, for regulatory tasks is authenticity. In other words, database query will exactly return the record that has been stored in the knowledgebase, whereas semantic similarity search may lead to similar but not exactly matching results. Thus, database query helps eliminate a major concern related to applying generative LLMs into the regulatory settings.

In AskFDALabel, we combined database query with an LLM by breaking down the whole information retrieval process of regular RAG into several steps. In some steps, such as template selection, entity recognition used an LLM to take advantage of its flexibility. For other steps such as labeling document retrieval, we decided to use a more conservative approach such as database query instead of a semantic similarity search, to ensure the retrieved reference would be relevant to the query, particularly when we knew the query was under a specific template.

In this study, we demonstrated that using such a hybrid information retrieval approach can provide promising result in all three tasks, which further indicated that combining an LLM with conventional technologies instead of relying on it alone could provide better and more explainable outcomes.

### Enhancing Regulatory Science with Modularized and Customizable Templates

In this study, we presented FDALabel-based RAG, which restricted the reference used in the LLM inference solely to the specific labeling documents instead of using the full scope of the LLM’s inherent knowledge. For example, we did not expect AskFDALabel to process such tasks as “rephrase my proposal,” or “suggest a travel plan for my weekend,” but only to focus on labeling document review and analysis.

Moreover, the AskFDALabel framework is designed to “plug-and-play” the foundation LLMs, like Llama 3.1-70B. One advantage of this strategy was the ability to directly benefit from the features introduced in the newer models, such as the increased token size newly introduced in Llama 3.1.

Along with directly using the foundation model, developing a fine-tuned LLM for such a specific task will be an option for future exploration. To do that, a large corpus of documents must be collected to train/fine-tune domain-specific LLMs, in addition to the demanding requirement of massive hardware resources. Currently, externally hosted LLMs such as ChatGPT from OpenAI and Gemini from Google are not allowed for use in regulatory research and review at the agency. When policy changes, they will be explored and compared with Llama in terms of efficiency, cost, and performance gain for the AskFDALabel framework.

### Integrating Human Expertise into the LLM Framework

In general, the manual reviewing process for labeling analysis can be divided into two steps. First, a strategy or protocol needs to be developed for the specific task. For example, for DILI classification, the reviewer needs to define which labeling section needs to be reviewed, the weight of different keywords in each section, and the severity level of DILI. After developing the strategy, the second step for reviewers is to undertake a labor-intensive process, such as manual document comparison or exhaustive database searches, to gather necessary information required for this task. Such processes might entail, for example, manually reading the labeling document and identifying and extracting all DILI-relevant keywords from section texts. This step is also prone to human errors or inconsistencies.

That said, AskFDALabel is designed to automate the second step, to augment the reviewers’ analytical capabilities. Our current experiments demonstrated that with appropriate templates and prompts, AskFDALabel can generate highly consistent and reproducible results for human reviewers, which will largely reduce the manual effort involved in implementing the designed annotation strategy in the second step. Particularly, the output of AskFDALabel can provide information beyond the final answer, such as, which labeling document (i.e., its set-id) was referred to, or which DILI-related keywords were found and used to determine the DILI classes. This additional information can help reviewers more efficiently check and validate the AskFDALabel output, and increases the reliability and trustworthiness of applying AskFDALabel to their daily tasks.

### Limitations and Future Directions

Our approach used fixed structured query language (SQL) queries to retrieve labeling documents by drug name, ensuring stability and data consistency. However, this static method limits adaptability for specific user inputs, such as routes of administration or dosage forms. Future work may incorporate LLMs to dynamically generate SQL queries from user prompts, though testing is required to confirm reliability.

Additionally, fine-tuning LLMs with domain-specific data is expected to enhance their performance in addressing regulatory-specific queries, but care must be taken to balance improvements with computational costs and potential risks of overfitting to narrow datasets.

While this study used only public drug labeling documents, AskFDALabel’s framework is adaptable for secure, non-public data, enabling comparisons across historical and bioequivalent documents. These promising results suggest broader potential for applying AI to confidential regulatory documents, such as safety reports and training materials, advancing AI’s role in regulatory science.

## Conclusion

AskFDALabel could be a game-changer since it has shown not only high accuracy and consistency, but also a capability to handle an ever-growing volume of data with a scalable and automated approach. Moreover, by providing additional explanations and cited references along with its answers, it can enhance the reliability of the automated process, making it easier for human reviewers to verify and trust the AI outputs. In all, we demonstrated that the generative AI could enhance the efficacy and effectiveness of drug safety research, particularly with, but possibly not limited to, drug labeling documents.

In conclusion, the integration of advanced natural language processing (NLP) techniques and LLM-powered systems like AskFDALabel represents a significant leap forward in the field of AE research and drug safety. By automating the labor-intensive processes of AE annotation, profiling, and classification, AskFDALabel not only enhances efficiency but also improves the accuracy and consistency of data extraction from FDA drug labeling documents. This innovation has the potential to transform regulatory practices and advance the understanding of AE annotation, profiling, monitoring, and drug toxicity classification, ultimately contributing to the safe and effective use of drugs for better patient outcomes. The evaluation of AskFDALabel across multiple benchmark datasets demonstrates its effectiveness, marking a promising step toward more reliable and scalable solutions in pharmacovigilance and drug safety research.

## Supplementary Information

Below is the link to the electronic supplementary material.

Supplementary file1 (PDF 164 KB)

Supplementary file2 (XLSX 14 KB)

Supplementary file3 (XLSX 35 KB)

Supplementary file4 (XLSX 132 KB)

Supplementary file5 (XLSX 374 KB)

## Declarations

### Availability of Data and Materials

The FDALabel database can be accessed from https://nctr-crs.fda.gov/fdalabel/ui/search. All data used in this study can be found in the electronic supplementary material (Online resources 3–5).

### Code Availability

The prompt and SQL query used in the study can be found in the electronic supplementary material (Online resource 1).
