# Supplementary material for: Leveraging FDA Labeling Documents and Large Language Model to Enhance Annotation, Profiling, and Classification of Drug Adverse Events with AskFDALabel
Source: Drug Saf. 2025 Feb 20;48(6):655–65. doi: 10.1007/s40264-025-01520-1 (PMC12098182; doi:10.1007/s40264-025-01520-1)
Supplement: Supplementary file 1 — Supplementary file1 (PDF 164 KB) [file 40264_2025_1520_MOESM1_ESM.pdf]

Leveraging FDA Labeling Documents and Large Language Model to Enhance Annotation, Profiling, and Classification of Drug Adverse Events with AskFDALabel

[1]Leihong Wu, [2]Hong Fang, [1]Yanyan Qu, [1]Joshua Xu, [1]Weida Tong,

[1]Division of Bioinformatics and Biostatistics, National Center for Toxicological Research, U.S. FDA, 3900 NCTR Rd, Jefferson AR, 72079

[2]Office of Scientific Coordination, National Center for Toxicological Research, U.S. FDA, 3900 NCTR Rd, Jefferson AR, 72079

Prompt used in specific templates

The prompts applied in specific templates and the LLM-based NER prompt for drug identification are described as below:

LLM-based NER

Extract any drug name from the given text.
After the drug was found, list all alias names.
For each synonym, add a tag <alias> around it. The output should only contain the drug name which can be searchable as an attribute in a database, no chemical or IUPAC name should be output. For example:
Input:
What are the Adverse Events reported for acetaminophen?'
Output:
<alias>Acetaminophen</alias>
<alias>APAP</alias>

Special Template determination

Given the input query from user, determine which template should be applied to process this query.
A template is specially designed process to fit the purpose of the given query. For example, if the query is for DILI classification, such as "what is the DILI class of abacavir?", it should use the template of "DILI Classification".
There are currently three templates in the system to choose:
[M1]Drug AE profiling: Generate Adverse Events (AEs) profiling of a given drug, based on its labeling document;
[M2]DILI Classification: Determine whether a drug can cause liver injury (Drug-Induced Liver Injury, DILI) based on its labeling document;
[M3]Biomarker Identification: ...
[M4]DICT Classification: Determine whether a drug can cause cardiotoxicity (Drug-Induced CardioToxicity, DICT) based on its labeling document;
if no special template can fit the given query, return using the generic mode as:
[G1]Generic Mode: if no special template is suitable for the given query.
Output guidance:
Note that the output should NOT answer the given query, but only provide which special template to use, or using the generic mode.
Example 1: Input:
What are the adverse events for Acetaminophen?
Example 1: Output:

[M1]Drug AE profiling
Example 2: Input:
What is the DILI class of Abacavir?
Example 2: Output:
[M2]DILI Classification
Example 2: Input:
What is the DICT class of tamoxifen?
Example 2: Output:
[M3]DICT Classification

DILI Classification

Drug Induced Liver Injury (DILI) is the topic of interest for your study on drug labeling document.
Check the given paragraph to see if any of the following DILI-relevant keywords (or with similar meaning) are mentioned in the paragraph.
if one keyword occurs multiple times in the paragraph, only list once. The output should contain the exact term mentioned in the content.
In addition, determine which severe level of DILI the keyword(s) reflect
Severity determination
The severity of Drug Induced Liver Injury should be determined by the following criteria and scores.
[Score: 8]Fatal liver failure. [Description] Hepatotoxicity: Death; fatal liver failure; or needed liver transplantation.
[Score: 7]Acute liver failure. [Description] Liver/hepatic failure; fulminant hepatic necrosis
[Score: 6]Liver necrosis. [Description] Histologically confirmed liver necrosis caused by drug; other types of ischemic or necrosis such as injection site necrosis should not count.
[Score: 5]Jaundice. [Description] Jaundice (clinically apparent), if caused by drug-induced hepatocellular injury
[Score: 4]Hyperbilirubinemia. [Description] Hyperbilirubinemia without visible jaundice, if not due to other causes like Gilbert syndrome or cholestasis
[Score: 3]liver/hepatic injury. [Description] Abnormal liver/hepatic function test; liver/hepatic injury; Liver-related aminotransferases increase (e.g. ALT, AST, transaminase, aminotransferase);
[Score: 2]Cholestasis; steatohepatitis. [Description] Steatohepatitis, cholestasis, cholestatic hepatitis; liver/hepatic damage/disorder/impairment/toxicity/reaction; hepatitis; hepatopathy
[Score: 1]Steatosis. [Description] Steatosis; fatty liver;
Output format (the answer should be in one line, below include multiple answer examples):
(Found) keyword1; keyword2; [Severity]: [Score: 8]Fatal liver failure
(Found) keyword1; keyword2; [Severity]: [Score: 3]liver/hepatic injury
(No Keywords) No DILI keyword was identified in the given paragraph

DICT Classification

Drug Induced Cardiotoxicity (DICT) is the topic of interest for your study on drug labeling document.
Check the given paragraph to see if any of the following DICT-relevant terms (or with similar meaning) are mentioned in the paragraph.
if one term occurs multiple times in the paragraph, only list once.
In addition, determine whether the keyword(s) reflect severe events of Drug Induced Cardiotoxicity
Severity determination
The severity of Drug Induced Liver Injury should be determined by the following criteria, including three levels (Severe, Moderate, Mild) on two aspects (Heart damage and Arrhythmia).
Severe(Heart damage)Fatal, life-threatening, death, need heart transplantation, (acute) myocardial infarction, heart attack, heart/cardiac failure, congestive heart failure (CHF), cardiomyopathy, myocarditis, coronary artery disease, myocardial ischemia, left ventricular dysfunction, cardiogenic shock, coronary artery insufficiency, valvular heart disease, endocarditis...
Severe(Arrhythmia) Fatal, life-threatening, heart/cardiac arrest, cardiorespiratory arrest, torsade de pointes (TdP), AV block III, ventricular fibrillation, Brugada syndrome…
Moderate(Heart damage) Angina pectoris, increased angina, mitral valve regurgitation, heart valve thickening, cardio spasm…
Moderate(Arrhythmia) Ventricular tachycardia, long QT syndrome/QT interval prolongation, ventricular arrhythmias…
Mild(Heart damage) Blood pressure (hypotension/hypertension) …
Mild(Arrhythmia) AV block I & II, atrial fibrillation, tachycardia/bradycardia, palpitations, sinus node dysfunction…

Also, cardiotoxicity terms may occur in the labeling for describing special conditions of the patient, such as "Patients with cardiac/heart disorder (ex. acute myocardial infarction…), hypersensitivity reactions, drug interactions, cardiac adverse events in elderly patients...". In such cases, its severity should be categoried as "Special Condition"

Output format (the answer should be in one line, below include multiple answer examples):
(Found) keyword1; keyword2; [Severity]: Severe(Heart damage)
(Found) keyword1; keyword2; [Severity]: Special Condition
(No Keyword) No DICT keyword was identified in the given paragraph

Drug AE Profile

Identify and Extract all reported Adverse Events from the given paragraphs.
The output should follow the format given below:
Example of Output:
The Adverse Events reported in this section are listed as below:
[AE1]: <context>
[AE2]: <context>

Semantic Match

Determine whether the below AE term/short phrases are mentioned in the given sentence. The tested AE term are:
(list-of-terms)
Output Format
If yes, return the original sentence with all relevant AE terms afterwards tagged with $$. If multiple terms were added, separate them with ;
If not, return "NOT RELATED"
Examples
…

SQL query for DILI/DICT classification

```
with table_filtered as (
            select *
              from sum_spl l
              join spl s on s.set_id = l.set_id
              where (REGEXP_LIKE(Upper(l.PRODUCT_NAMES), {name_list})
              or REGEXP_LIKE(Upper(l.PRODUCT_NORMD_GENERIC_NAMES),
{name_list}))
        )
        (select *
              from table_filtered
              where document_type_loinc_code in ('34391-3', '45129-4')
        UNION ALL
        select *
              from table_filtered
              where document_type_loinc_code in ('34390-5')
              AND NOT EXISTS (
                select 1
                  from table_filtered
                  where document_type_loinc_code in ('34391-3', '45129-4')
              )
        )
        order by eff_time desc;
```
